# Learning From Neighbors

**DOI:** 10.9745/GHSP-D-20-00639

**Published:** 2020-12-23

**Authors:** Stephen Hodgins

**Affiliations:** a Editor-in-Chief, Global Health: Science and Practice Journal, and Associate Professor, School of Public Health, University of Alberta, Edmonton, Alberta, Canada.

## Abstract

We can learn valuable lessons from program efforts that at first glance may seem to be far removed from our own work.

See related article by Duttine et al..

The article by Duttine and colleagues^1^ in this issue of *Global Health: Science and Practice* describes efforts recently undertaken in Brazil to develop and test an approach to supporting parents of young children affected by Zika. For the vast majority of GHSP readers, we suspect that this topic is not directly relevant to their work. Nevertheless, we believe we can learn valuable lessons from program efforts that may seem, at first glance, to be far removed from our own work.

The authors describe their process in the development and initial testing of a community-based support program for parents. Their starting points were: (1) *a clearly defined need*, and (2) a *potentially relevant model*. The need was for effective, formalized support of caregivers of community-living children with mild to moderate Zika-related impairments. The model was a caregiver education and support program first developed in Bangladesh and subsequently adopted in many other countries: Getting to Know Cerebral Palsy.

With this identified gap and a potential solution, the next step was a *needs assessment*, which included a review of relevant literature and consultation with stakeholders, both experts and parents of affected children. On the basis of this input, the developers then crafted an *initial version of the program design and materials* (adapted to Zika and to a Brazilian cultural setting) and an associated *theory of change* (a theory of how they thought the intervention would work).

The program developers then tested the program in 2 diverse sites, going through 2 *iterative rounds of piloting* to assess relevance, usefulness, and feasibility. Adaptation and refinement of the program was done not only at the end of each of these rounds; the developers also pursued an intentional approach of *fast-track learning*—eliciting feedback on an ongoing basis from participants and facilitators and making real-time changes, as necessary, in content, approach, and logistical arrangements.

As they explain, not only was the program itself iteratively adapted and modified, so too was the theory of change. In the course of trying out the intervention, the investigators formed a better sense of how it worked and, in turn, made revisions to their theory of change.

The authors modeled *learning from neighbors*, drawing on an approach developed in another context for a somewhat different need (Bangladeshi families with children affected by cerebral palsy) and adapting it to their setting to address their specific problem. We encourage you—our readers—also to learn from neighbors, drawing from examples like this of how to address a public health problem by:

- Listening to stakeholders
- Being flexible and prepared to revisit assumptions and early design choices
- Building learning and adaptation into your routine ways of doing business

## References

[1] Duttine A , Smythe T , Ribeiro Calheiros de Sa M, Ferrite S, Moreira ME, Kuper H. Juntos: a support programme for families impacted by congenital Zika syndrome in Brazil. Glob Health Sci Pract. 2020;8(4). 10.9745/GHSP-D-20-00018 PMC7784063 33361247

